# Effectiveness of smoking cessation intervention based on the ABC Approach in patients with TB

**DOI:** 10.5588/pha.23.0057

**Published:** 2024-06-01

**Authors:** A. Ohkado, A. Querri, J. Bermejo, R. Bartolome, G. Pardilla, D. Manese, J. Recidoro, L. Kawatsu, A.M.C. Garfin, T.S. Bam

**Affiliations:** ^1^ Department of Epidemiology and Clinical Research, Research Institute of Tuberculosis (RIT), Japan Anti-Tuberculosis Association (JATA), Kiyose, Japan;; ^2^ Manila Health Department (MHD), Manila, The Philippines;; ^3^ School of Nursing, Nagoya City University, Nagoya, Japan;; ^4^ Department of Health, Disease Prevention and Control Bureau (DOH-DPCB), Manila, The Philippines;; ^5^ The UNION Asia Pacific Office, Singapore

**Keywords:** tobacco, passive smoking, treatment, tuberculosis, The Union, The Philippines

## Abstract

**SETTING:**

Urban setting in the Philippines.

**OBJECTIVE:**

To assess the effectiveness of the ABC Approach developed by The Union as a tobacco-smoking cessation intervention for TB patients at a primary healthcare level in an urban setting in the Philippines.

**DESIGN:**

We set up an intervention group whose patients with TB received the ABC approach and a control group of patients with TB receiving only routine health education in Manila, The Philippines. We collected smoking status and the domestic secondhand-smoking (SHS) status data from patients with TB at months 0, 2, 4, 6, 8, and 12. TB treatment outcome data were also collected.

**RESULTS:**

Patients with TB (*n =* 2,174) were enrolled upon TB registration. Smoking rates were consistently low in the intervention group (3.9% vs. 8.7% at Month 6). The odds ratios of both tobacco-smoking status and domestic SHS status in the intervention group were significantly lower than those in the control group (tobacco-smoking status: *P* < 0.001, domestic SHS status: *P* < 0.01). TB treatment success rates were similar between the groups (85.0% vs. 87.3%; *P* = 0.201).

**CONCLUSION:**

The ABC approach successfully reduced tobacco-smoking rates, maintained low domestic SHS rates and TB treatment success rates in the Philippines.

## Abstract (fr)

**CADRE:**

Cadre urbain aux Philippines.

**OBJECTIF:**

Évaluer l'efficacité de l'approche ABC développée par L'Union en tant qu'intervention de sevrage tabagique pour les patients atteints de TB au niveau des soins de santé primaires en milieu urbain aux Philippines.

**MÉTHODE:**

Nous avons mis en place un groupe d'intervention dont les patients atteints de TB ont reçu l'approche ABC et un groupe témoin de patients atteints de TB ne recevant qu'une éducation sanitaire de routine à Manille, aux Philippines. Nous avons recueilli des données sur le statut tabagique et le statut de tabagisme passif (SHS, pour l’anglais « *secondhand-smoking* ») domestique des patients atteints de TB aux mois 0, 2, 4, 6, 8 et 12. Des données sur les résultats du traitement de la TB ont également été recueillies.

**RÉSULTATS:**

Les patients atteints de TB (*n =* 2 174) ont été recrutés lors de l'enregistrement de la TB. Les taux de tabagisme étaient constamment faibles dans le groupe d'intervention (3,9% contre 8,7% au 6e mois). Les rapports de cotes du tabagisme et du statut SHS canadien dans le groupe d'intervention étaient significativement inférieurs à ceux du groupe témoin (tabagisme : *P* < 0,001, statut SHS national : *P* < 0,01). Les taux de réussite du traitement de la TB étaient similaires entre les groupes (85,0% contre 87,3% ; *P* = 0,201).

**CONCLUSION:**

L'approche ABC a permis de réduire les taux de tabagisme, de maintenir de faibles taux nationaux de SHS et de taux de réussite du traitement de la TB aux Philippines.

The WHO estimates that around 10.6 million people have TB worldwide, killing approximately 1.3 million people annually.^1^ TB remains a considerable health burden in the Philippines, with an estimated 638 TB incident patients per 100,000 population in 2022.^1^ In comparison, tobacco smoking is the most substantial single cause of death worldwide, estimated to cause more than eight million deaths annually.^2^ Tobacco smoking is a critical risk factor for many non-communicable diseases and puts family members at similar health risks.^3^ A report estimated that the number of smokers in the Philippines was approximately 17.3 million; the total and male smoking rates were 28.3% and 47.6%, respectively.^4^ Tobacco smoking is also a risk factor for active TB, poor TB treatment outcomes, relapse, and TB mortality.^5–14^ Considering the severe effects of tobacco smoking on TB control, The Union Lung Health Scientific Section developed the ABC (A = ask, B = brief advice, C = cessation support) approach in 2010.^15^ The ABC approach has been piloted and tested within a regular TB control mechanism in several countries, such as Bangladesh,^16^ Pakistan,^17^ China,^18^ Nepal,^19^ Indonesia,^20^ Sudan,^21^ and South Africa.^22^

Primary healthcare workers are expected to provide health education, including tobacco smoking cessation, to all TB patients who smoke as part of their routine health promotion activities in the Philippines. National guidance still needs to be developed to integrate tobacco smoking cessation systemically with TB control in the Philippines.

This pilot study aimed to assess the effectiveness of an ABC approach developed by The Union as a tobacco-smoking cessation intervention at the primary healthcare level in an urban setting in the Philippines.

## STUDY POPULATION, DESIGN, AND METHODS

This pilot intervention study collected smoking status data from the patients with TB upon registration and at months 2, 4, 6, 8, and 12 (Supplementary Data shows the definition of terms in more detail; see Supplementary Table S1A and Supplementary Table S1B in Supplementary Data 1). The participants were all patients with TB, including those aged ≥18 years, with no multidrug-resistant TB, and those who submitted informed consent forms to participate in the study. We collected age upon TB registration, sex, TB disease site, TB category, comorbidities, and sputum smear examination results from the National Tuberculosis Control Programme (NTP) treatment card and TB patient register. In addition, we collected information on education level, current marital status, occupation, and economic status from all patients with TB enrolled upon TB registration. TB treatment outcome data were collected from all patients enrolled during the study period.

We established an intervention group (Group I) in which patients with TB received the ABC Approach as an intervention.^15^ The control group (Group C) participants received only routine health education. We conducted a one-day training cessation on the ABC Approach for all health staff in the intervention group and a half-day orientation cessation for all health staff in the control group. The patient flows of Groups I and C are indicated in Supplementary Figures S1A and S1B (see Supplementary Data 2).

We set District I in Manila as Group I. This Group had a population of approximately 410,000, comprised of 10 health centres with about 500 bacteriologically confirmed and 500 clinically diagnosed TB cases per year. We set District VI in Manila as Group C. This Group had an approximately 260,000 population, comprised of ten health centres with about 250 bacteriologically confirmed TB cases and 450 clinically diagnosed TB cases per year.

We assumed a lower tobacco-smoking rate at the end of the TB treatment in Group I than in Group C, i.e., 20% vs. 30%, with a significance level of 0.05 and a power to detect the difference of 0.8. The sample size required for each group was at least 294 patients with TB who smoked. Assuming that we would lose 10% of the sample population, the sample size of each group required was 323 patients with TB who smoke. Consequently, the sample size needed for each group was 923 patients with TB, assuming that the smoking rates upon TB registration of both groups were 35%. The health staff at each health centre offered the ABC Approach for 5–10 min within the standard TB patient service at each health centre in District I, Manila.

We implemented two data validation mechanisms for the tobacco-smoking status of TB patients and their domestic secondhand-smoking (SHS) status. Namely, the closest family members of TB patients were asked (interviewed) by health workers at the health centre to validate their tobacco-smoking and domestic SHS status. In addition, the community health volunteers, who routinely conduct home visits, validated the tobacco-smoking and domestic SHS status on their routine home visits. No biophysiological smoking status validation tools were used because they were unsustainable as validation tools in practice.

The key indicators we set in the study were tobacco smoking rate, tobacco cessation rate, domestic SHS rate, and TB treatment success rate. We have set the definitions of terms related to tobacco smoking status in this study in Supplementary Tables S1A and S1B (Supplementary Data 1).

### Statistical analyses

We applied the χ^2^ or Fisher’s exact test for statistically significant differences between categorical data. We performed multivariate logistic regression analysis for the dependent variables. We adjusted for possible independent variables such as age upon registration, sex, TB disease site, TB category, comorbidities, education level, current marital status, occupation, and economic status. We also calculated E-values to assess the unmeasured confounding factors that could affect the regression analysis for each dependent variable as a sensitivity analysis.^23–25^ *P* < 0.05 was considered statistically significant.

### Ethical considerations

Relevant guidelines and regulations were applied to all methods. Written informed consent was obtained from all subjects or their legal guardian(s). The institutional review boards approved the study protocol at Jose R. Reyes Memorial Medical Center, Manila, the Philippines (IRB Protocol No.2016-101) and the Research Institute of Tuberculosis, Tokyo, Japan (RIT/IRB 28-16).

## RESULTS

### Participants’ characteristics

**FIGURE 1.**
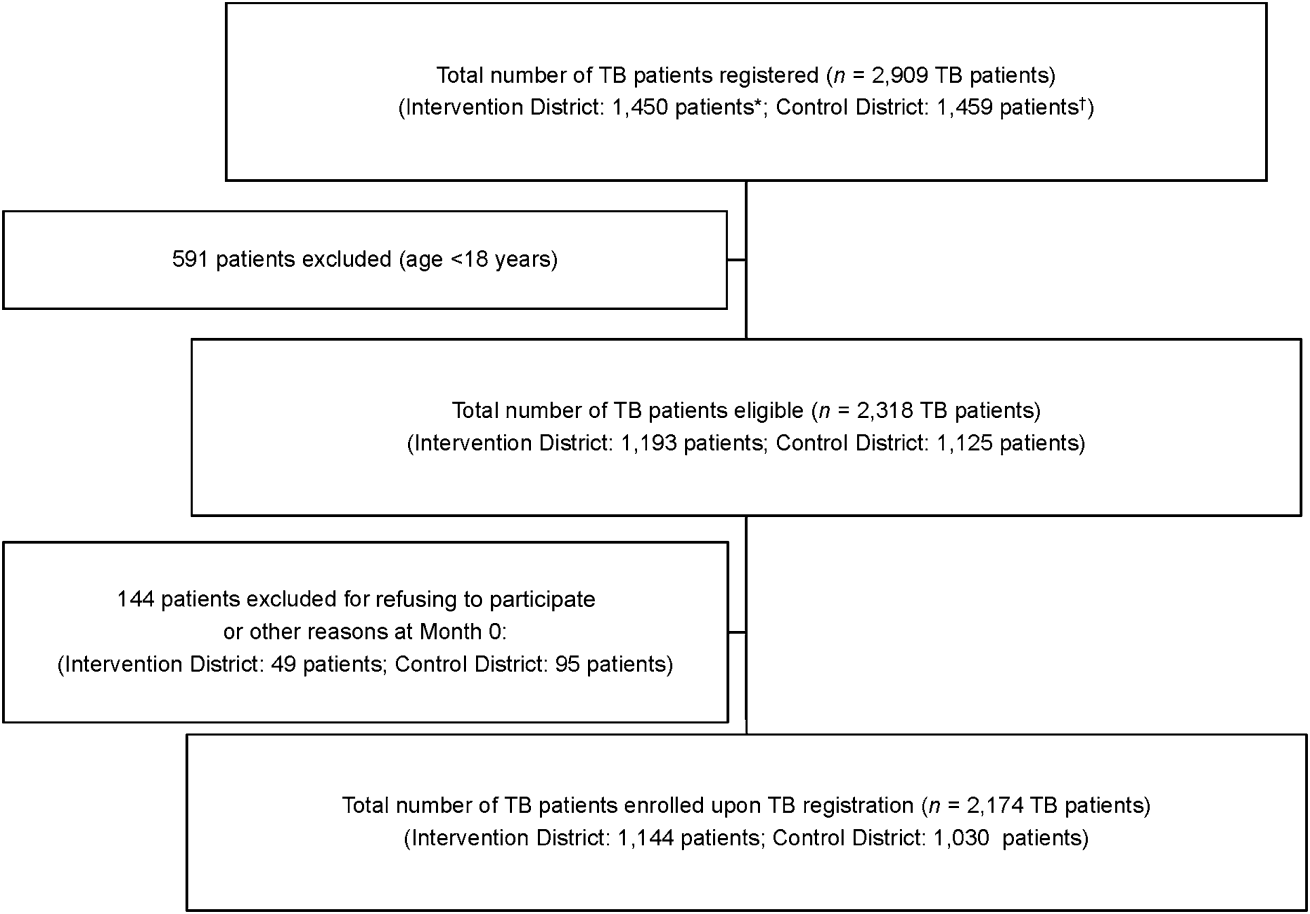
TB patients’ enrolment flow at Month 0 of TB registration, Manila, the Philippines. *The Intervention District (District I in Manila) had 10 health centres. ^†^The Control District (District IV in Manila) had 10 health centres.

The total number of patients with TB registered from April 2017 to March 2018 in District I (Group I) and from April 2017 to October 2018 in District VI (Group C), Manila, the Philippines, was respectively 1,450 and 1,459 patients. After excluding the patients with TB who did not meet the enrolment criteria, 2,174 TB patients, i.e., 1,144 in District I (Group I) and 1,030 in District VI (Group C), were enrolled upon TB registration. Figure 1 gives the study flow chart of participants at Month 0 of TB registration.

### Tobacco-smoking status

The profiles of patients with TB enrolled in Month 0 of the study are given in Supplementary Table S2 (Supplementary Data 3). Group I had a higher proportion of those with a patient delay of ≥30 days (61.0% vs. 52.5%; *P* < 0.001) and with cough (83.1% vs. 68.0%; *P* < 0.001); a lower proportion of those with a high education history (19.8% vs. 26.8%; *P* = 0.001) compared with those of Group C.

The point prevalence of tobacco-smoking status at Months 0–12 is indicated in the Table. The tobacco-smoking rate in Group I at Month 0 was slightly higher than that in Group C but was statistically insignificant (31.6% vs. 27.3%, *P* = 0.074). The tobacco cessation rates in Group I from Months 2 to 12 were consistently high. In contrast, tobacco smoking rates in Group I were consistently lower than those in Group C.

**TABLE.** Point prevalence of tobacco-smoking status of patients with TB by month of TB registration, the Philippines.*

	Intervention Group: District I, Manila	Control Group: District VI, Manila	*P*-value^†^
	*n* (%)	*n* (%)	
Month 0 (*n* = 2,174)	(*n* = 1,144)	(*n* = 1,030)	0.074
Non-smokers	783 (68.4)	749 (72.7)	
Ex-smokers	236 (20.6)	214 (20.8)	
Never-smokers	547 (47.8)	535 (51.9)	
Current smokers	361 (31.6)	281 (27.3)	
Month 2 (*n* = 2,096)	(*n* = 1,100)	(*n* = 996)	<0.001
Quitters	274 (24.9)	157 (15.8)	
Temporary quitters	194 (17.6)	127 (12.8)	
Permanent quitters	80 (7.3)	30 (3.0)	
Non-smokers	711 (64.6)	715 (71.8)	
Smokers	61 (5.6)	112 (11.2)	
Current smokers	58 (5.3)	107 (10.7)	
Relapsed	3 (0.3)	5 (0.5)	
Others/unknown	54 (4.9)	12 (1.2)	
Month 4 (*n* = 1,979)	(*n* = 1,013)	(*n* = 966)	<0.001
Quitters	274 (27.1)	166 (17.2)	
Temporary quitters	54 (5.3)	45 (4.7)	
Permanent quitters	220 (21.7)	121 (12.5)	
Non-smokers	691 (68.2)	708 (73.3)	
Smokers	45 (4.4)	92 (9.5)	
Current smokers	40 (4.0)	89 (9.2)	
Relapsed	5 (0.5)	3 (0.3)	
Others/unknown	3 (0.3)	0 (0.0)	
Month 6 (*n* = 1,967)	(*n* = 1,021)	(*n* = 946)	<0.001
Quitters	269 (26.4)	159 (16.8)	
Temporary quitters	25 (2.5)	20 (2.1)	
Permanent quitters	244 (23.9)	139 (14.7)	
Non-smokers	648 (63.5)	691 (73.0)	
Smokers	40 (3.9)	82 (8.7)	
Current smokers	38 (3.7)	82 (8.7)	
Relapsed	2 (0.2)	0 (0.0)	
Others/unknown	64 (6.3)	14 (1.5)	
Month 8 (*n* = 1,919)	(*n* = 991)	(*n* = 928)	<0.001
Quitters	212 (21.4)	153 (16.5)	
Temporary quitters	12 (1.2)	6 (0.7)	
Permanent quitters	200 (20.2)	147 (15.8)	
Non-smokers	527 (53.2)	678 (73.0)	
Smokers	28 (2.8)	89 (9.6)	
Current smokers	25 (2.5)	87 (9.4)	
Relapsed	3 (0.3)	2 (0.2)	
Others/unknown	224 (22.6)^‡^	8 (0.9)	
Month 12 (*n* = 1,878)	(*n* = 971)	(*n* = 907)	<0.001
Quitters	258 (26.6)	149 (16.4)	
Temporary quitters	6 (0.6)	2 (0.2)	
Permanent quitters	252 (26.0)	147 (16.2)	
Non-smokers	655 (67.5)	672 (74.1)	
Smokers	41 (4.2)	83 (9.2)	
Current smokers	40 (4.1)	81 (8.9)	
Relapsed	1 (0.1)	2 (0.2)	
Others/unknown	17 (1.8)	3 (0.3)	

* Definitions:

*Non-smoker:* A patient who has never smoked tobacco (Never smoker) or who used to smoke tobacco but has not smoked in the last 3 months (Ex-smoker).

*Never smoker:* A patient who has never smoked tobacco, not even a puff.

*Ex-smoker:* A patient at enrolment who used to smoke tobacco but has not smoked in the last 3 months, not even a puff.

*Current smoker:* 1) A patient at enrolment who has smoked in the last 3 months, even a puff. *OR* 2) A patient at a follow-up visit who has smoked in the last 2 weeks, even a puff, and has not attempted to quit (for at least 24 hours) since the last visit.

*Quitter:* A smoker who has temporarily quit tobacco or has remained committed to quitting. The quitter can be either a “Temporary quitter” or a “Staying quitter”.

*Temporary quitter:* A smoker who has quit tobacco for less than three months, including a smoker at baseline who has not smoked at all, even a puff, in the last 2 weeks during follow-up visits.

*Staying quitter:* A smoker who has remained tobacco-free for ≥3 months.

*Relapsed:* A smoker at baseline who has tried to quit during the ABC intervention but has relapsed (smoked in the last 2 weeks before the current visit) and has made at least one quit attempt lasting at least 24 hours since the last visit.

† For Month 0, the χ^2^ test was applied to each proportion of ex-smokers, never-smokers, and current smokers. For Months 2 through 12, the χ^2^ test was applied to each proportion of quitters, non-smokers, smokers, and others/unknown.

‡ 172 new TB patients without smoking status data were included.

**FIGURE 2.**
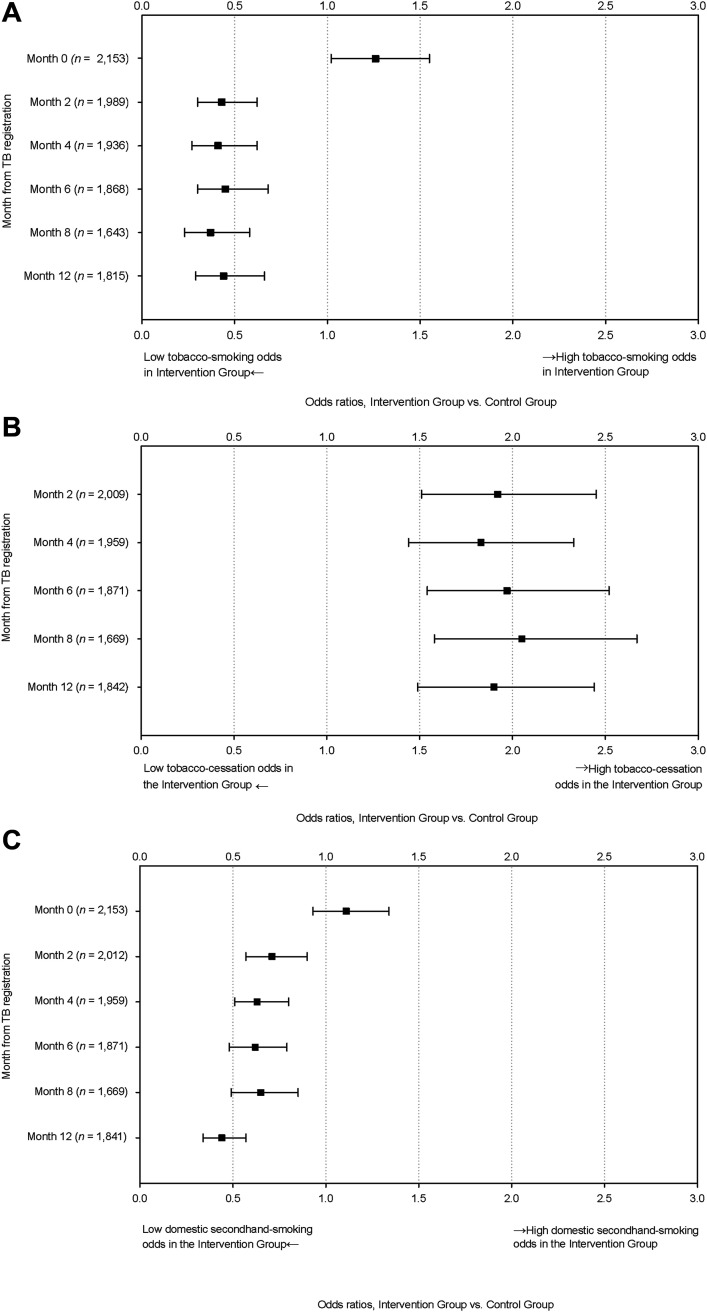
Odds ratios of **A)** tobacco-smoking status, **B)** tobacco-cessation status, and **C)** domestic secondhand-smoking status, with logistic regression analysis^*^ between Intervention District^†^ and Control District,^†^ Manila City, The Philippines. ^*^Binominal logistic regression analysis adjusted to the site, sex, age category, patient category, TB type, patient delay, education level, marital status, occupation, regular incomes, monthly household incomes, and HIV & DM status. Current smoking status, i.e., the dependent variable, was assigned 1 if the participant was either a current smoker or relapsed; otherwise, non-smokers, quitters, ex-smokers, or unknown was assigned 0. ^†^Intervention District: District I, Manila City, the Philippines, assigned as an intervention group; Control District: District VI, Manila City, the Philippines, assigned as a control group.

Figure 2A and Supplementary Table S3 (Supplementary Data 4) indicate the tobacco-smoking status using logistic regression analysis. The odds ratio (OR) of tobacco smoking in Group I at Month 0 was slightly higher than that in Group C (OR 1.26, 95% confidence interval [CI] 1.02–1.55). The ORs of tobacco smoking rates in Group I to those in Group C were consistently and significantly low from Months 2 to 12. The E-values from Months 2 to 12 showed consistently high point estimates and CIs.

Figure 2B and Supplementary Table S4 (Supplementary Data 5) indicate the ORs of tobacco cessation status using logistic regression analysis. The ORs of tobacco cessation in Group I to that in Group C were consistently higher from Months 2 to 12.

### Domestic secondhand-smoking status

Supplementary Table S5A (Supplementary Data 6) indicates the domestic SHS status by months from TB registration by the site in Manila from 2017 to 2018. There was no significant difference in domestic SHS rate between Groups I and C at Month 0, but it was consistently lower in Group I than in Group C from Months 2 through 12. Logistic regression analysis for domestic SHS status between the sites indicated significantly and consistently lower ORs of Group I to those of Group C from Months 2 to 12, whereas no significant difference was observed at Month 0, as shown in Figure 2C and Supplementary Table S5B (Supplementary Data 6).

### TB treatment outcomes

Supplementary Table S6A (Supplementary Data 7) shows the TB treatment outcomes among 2,246 patients with TB. The total number of patients with TB with treatment outcomes increased from the total number enrolled at Month 0 because some patients with TB enrolled after Month 0. There was no significant difference in the TB treatment success rate between the sites (85.0% vs. 87.3%, *P* = 0.201). The OR of TB treatment success at the intervention site to that at the control site was not significantly different (*P* = 0.477, Supplementary Table S6B in Supplementary Data 7).

## DISCUSSION

The ABC Approach by The Union successfully reduced tobacco-smoking rates, kept tobacco-smoking cessation rates high, and kept the domestic SHS rates low for TB patients during TB treatment and at Month 12 of TB registration in an urban setting in the Philippines. While in the control district, the tobacco-smoking rates were also reduced, but the tobacco smoking cessation rates were low and the domestic SHS rates were high. This finding agrees with a report on follow-up smoking cessation intervention after 5 years, which showed a higher non-smoking rate among TB patients who received cessation intervention in China (43.5% vs. 30.0%).^26^ The present study just followed up on the smoking status of TB patients up until 12 months after TB registration; we are still determining if we will be able to see similar findings in China in 5 years.

Taking the currently available evidence about the effectiveness of brief smoking cessation interventions, Chiang & Bam insisted on implementing the interventions for all patients with TB more widely with the minimum condition of smoke-free health facilities.^27^ Shin et al. also insisted on implementing smoking cessation interventions for patients with TB with smoke-free policies because “exposure to smoking at health facilities will inhibit the patient’s smoke quitting attempts”.^28^

We did not detect any significant difference in TB treatment success rates between the intervention and control districts, i.e., 85.0% vs. 87.3%, respectively. This finding does not align with that reported from Sudan, indicating a much better treatment success rate among the enrolled TB patients than among those who did not, i.e., 83% and 59%, respectively.^21^ In contrast, a report from North India indicated that TB treatment success was lower in the intervention arm than in the control arm, although it was not statistically different.^29^ A systematic review reported in 2016 concluded that it was inconclusive whether smoking cessation interventions improve TB treatment outcomes.^30^ The TB treatment success rates in the intervention and control districts of the present study were 87% and 91% in 2017 before the current intervention study started, respectively. Our findings indicate that the ABC Approach did not at least negatively affect TB patient care regarding achieving TB treatment success.

One of the limitations of the present study is that we did not use any biophysiological measurement tools to measure urine cotinine^31^ or exhaled carbon monoxide levels^32^ to strictly verify the tobacco-smoking status of the study participants. This may have caused a misclassification bias because current smokers tend to under-self-report their smoking status.^33^ The results may have underestimated the participants' smoking status despite our efforts to minimise the potential misclassification bias using routine home visits by community health volunteers to verify the smoking status. Nonetheless, the tobacco-smoking rate at Month 0 in the intervention district was significantly higher than that in the control district with an odds ratio of 1.26 (*P* = 0.032), and the tobacco-smoking rates markedly dropped in the intervention district at Month 2. Second, we implemented smoking cessation interventions at the primary healthcare level in an urban setting in the Philippines. Hence, we cannot extrapolate the findings to other locations. Third, the present study did not randomly assign the intervention and control districts; hence, some unmeasurable confounding factors may have affected smoking and domestic SHS status, TB treatment outcomes (dependent variables), and exposure variables. Nonetheless, the E-values related to the tobacco-smoking status from Months 2 to 12 indicated relatively high point estimates with confidence intervals, suggesting fairly robust logistic regression analysis results on the tobacco-smoking status. Fourth, we focussed on tobacco smoking and excluded novel tobacco products such as nicotine-containing e-cigarettes or heated tobacco products. Therefore, we need to interpret the present study findings cautiously when expanding our conclusions to interventions for novel tobacco products.

Our study presents several important points. First, we implemented routine smoking cessation interventions at the primary healthcare level in the Philippines. Hence, sustaining the interventions routinely is highly possible without substantial investment. This is a primary reason for not utilising any biophysiological measurement tools to verify smoking status, which seemed irrelevant in a typical setup in the Philippines. Second, we set up intervention and control groups to test the effectiveness of smoking cessation interventions by comparing the results.

## CONCLUSIONS

In conclusion, the ABC Approach recommended by The Union was successfully implemented to reduce the tobacco-smoking rate, to maintain a high tobacco cessation rate and a low domestic SHS rate while maintaining good TB treatment outcomes in an urban setting in the Philippines. The National TB Control Programme, in collaboration with the tobacco-control programme in the Philippines, should expand the ABC Approach to save lives from the combined harms of TB and tobacco smoking.

## Supplementary Material


