# Supplementary material for: Effectiveness of smoking cessation intervention based on the ABC Approach in patients with TB
Source: Public Health Action. 2024 Jun 1;14(2):45–50. doi: 10.5588/pha.23.0057 (PMC11216290; doi:10.5588/pha.23.0057)
Supplement: Supplementary file 1 [file iutld_pha_23.0057_supplementarydata1.pdf]

http://dx.doi.org/10.5588/pha.23.0057

Effectiveness of smoking cessation intervention based on the ABC Approach in patients with TB

Supplementary Table S1A Definition of A, B, and C used for the ABC approach modified from reference 15 for the study

Abbreviation	Modified definitions
A	Ask about the smoking behaviour of TB patients and their family members via a face-to-face interview by health centre staff at each visit of the TB patients.
B	Brief advice, which includes personalized and general information offered at the clinic by the health centre staff at each visit of the TB patients. General advice is provided to TB patients who are smokers and non-smokers. If their family members accompanied them, the family members receive similar advice. If not, we instructed TB patients to provide advice to their family members to maintain a secondhand smoke-free home.
C	Cessation support is provided along with brief advice at each visit of the TB patients. TB patients who smoke and are ready to quit smoking are advised to 1) tell family, friends, and colleagues that they are quitting smoking; 2) remove smoking accessories from home and workplaces; 3) make their home smoke-free and avoid secondhand smoke; and 4) be given health education leaflets, pamphlets, and “No Smoking” signage to display at home.

Supplementary Table S1B Definition of terms used in this study, modified from reference 15

Terms	Definitions
**Domestic secondhand-smoking free**	A house with no evidence of tobacco smoking at the home visit by the investigators.
**Non-smoker**	A patient who has never smoked tobacco (Never smoker) or who used to tobacco smoke but has not smoked in the last three months (Ex-smoker)
**Never smoker**	A patient who has never smoked tobacco, not even a puff before.
**Ex-smoker**	A patient at enrolment who used to tobacco smoke but has not smoked in the last three months, not even a puff.
**Current smoker**	1) A patient at enrolment who has smoked in the last three months, even a puff or<br>2) A patient at follow-up visit who has smoked in the last two weeks, even a puff and has not attempted to quit (for at least 24 hours) since the last visit.
**Quitter**	A smoker who has quit tobacco temporarily or has remained to quit. The quitter is either a "Temporary quitter" or a "Staying quitter".
**Temporary quitter**	A smoker who has quit tobacco for less than three months, including a smoker at baseline who has not smoked at all, even a puff, in the last two weeks at the follow-up visits.
**Permanent quitter**	A smoker who remained tobacco-free for three months or more.
**Relapsed**	A smoker at baseline who has tried to quit during the ABC intervention but has relapsed (has smoked in the last two weeks before the current visit but has made at least one quit attempt lasting at least 24 hours since the last visit).
**Lost-to-follow-up**	A patient with status who did not attend the follow-up visit.
**Died**	A patient who has died of any cause during anti-TB treatment.

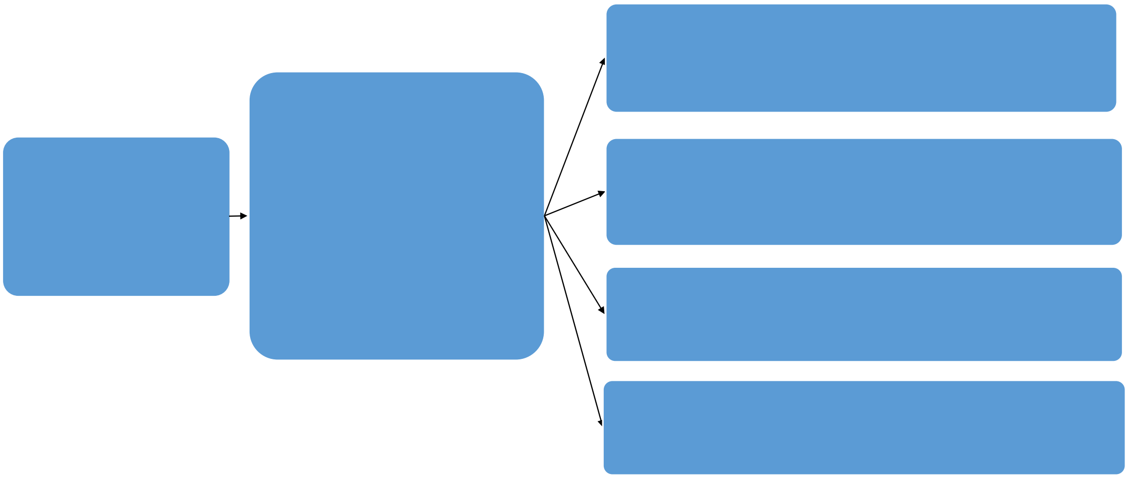

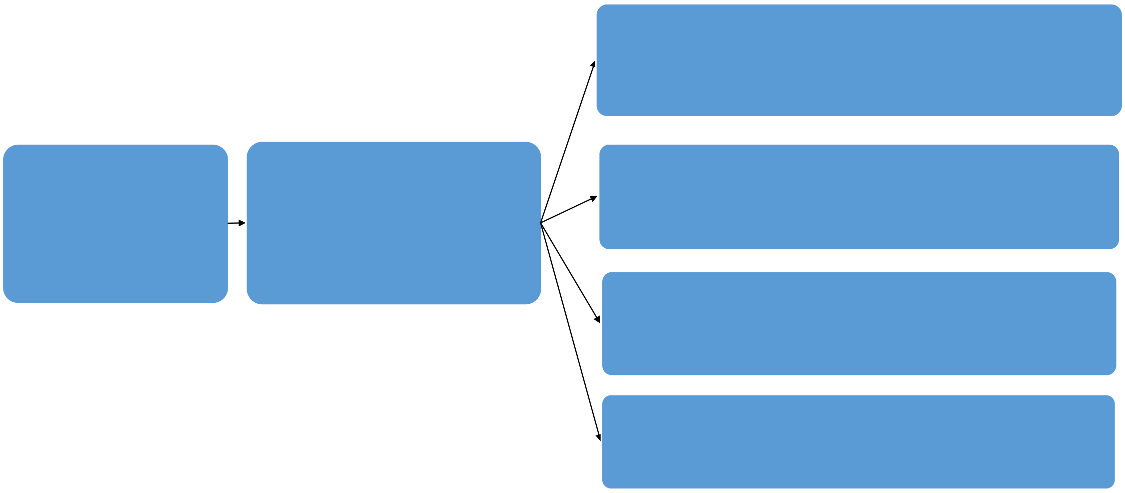

TB: tuberculosis

*1 Tobacco-smoking status was classified as one of the categories of “never smoker”, “ex-smoker”, “current smoker”, “temporary quitter”, “staying quitter”, or “relapsed.”

*2 Domestic secondhand-smoking status was defined as either secondhand smoke-free home or not.

*3 TB treatment outcomes were defined following the National TB Control guidelines in the Philippines. TB treatment success was defined as either “cured” or “treatment completed.”

*4 Post-TB treatment health status was self-reported as “well” or “unwell”.

Supplementary Table S2 TB patient profiles enrolled in the study upon TB registration, by the district in Manila, from 2017 to 2018, the Philippines, N=2,174

	Intervention district (Group I) (District I, Manila) n=1,144 (%)	Control district (Group C) (District VI, Manila) n=1,030 (%)	*p*-value *5
**Males**	713 (62.3)	645 (62.6)	0.877
=< 10 cigarettes	248 (68.9)	191 (68.2)	
11 – 20 cigarettes	90 (25.0)	80 (28.6)	
20 cigarettes <	22 (6.1)	9 (3.2)	
**Age groups**			0.987
18-29 years old	308 (26.9)	277 (26.9)	
30-49	372 (32.5)	335 (32.5)	
50-69	391 (34.2)	356 (34.6)	
70-	73 (6.4)	62 (6.0)	
**TB Patient category**			0.02
New	814 (71.2)	778 (75.5)	
Retreatment	253 (22.1)	208 (20.2)	
others	77 (6.7)	44 (4.3)	
**TB patient delay *1**			<0.001
30 days or less	445 (39.0)	482 (47.5)	
31 days or more	697 (61.0)	533 (52.5)	
**Symptoms upon TB registration**			
Cough	951 (83.1)	700 (68.0)	<0.001
Fever	208 (18.2)	224 (21.8)	0.037
Chest pain	78 (6.8)	106 (10.3)	0.139
Haemoptysis	67 (5.9)	79 (7.7)	0.092
Dyspnea	79 (6.9)	70 (6.8)	0.920
**Education History**			0.001
No formal education	12 (1.1)	14 (1.4)	

Elementary or secondary school level	894 (78.2)	728 (70.7)	
Post-secondary or college and above level	226 (19.8)	276 (26.8)	
Others or unknown	12 (1.1)	12 (1.2)	
**Marital status**			0.817
Never married	416 (36.4)	371 (36.0)	
Married or live together	594 (51.9)	546 (53.0)	
Widow/widower or separated/divorced	133 (11.6)	111 (10.8)	
Others or unknown	1 (0.10)	2 (0.19)	
**Occupations**			0.002
Self-employed	197 (17.2)	114 (11.1)	
Government employed	40 (3.5)	40 (3.9)	
Private company employed	192 (16.8)	167 (16.2)	
Day laborer	73 (6.4)	74 (7.2)	
Housekeeper	17 (1.5)	30 (2.9)	
Unemployed	519 (45.4)	497 (48.3)	
Student	53 (4.6)	61 (5.9)	
Others or unknown	53 (4.6)	47 (4.6)	
**Self-claimed regular home incomes** *2			0.034
	456 (40.0)	365 (35.4)	
**Self-claimed monthly household income** *3			<0.001
<4,000 Philippines pesos (PP)	224 (19.6)	109 (10.6)	
4,000 – 7,999 PP	310 (27.1)	254 (24.8)	
8,000 – 11,999 PP	285 (24.9)	274 (26.7)	
12,000 – 15,999 PP	153 (13.4)	176 (17.2)	
>=16,000 PP	172 (15.0)	213 (20.8)	
**Tobacco smoking dependency status among current smokers** *4 as the time until the first tobacco smoke after wake up			0.865

30 minutes or less	118 (32.8)	90 (32.1)	
More than 30 minutes	242 (67.2)	190 (67.9)	
**Tobacco smoking dependency status among current smokers** [*4] as the number of cigarettes smoked per day			0.181
=< 10 cigarettes	248 (68.9)	191 (68.2)	
11 – 20 cigarettes	90 (25.0)	80 (28.6)	
20 cigarettes <	22 (6.1)	9 (3.2)	

TB: tuberculosis

*1 17 patients were excluded due to missing data. Days between the onset of the TB symptoms and the first consultation with a health facility.

*2 The number indicates the sum of participants who claimed “yes.”

*3 Four patients were excluded due to missing data. One thousand pesos are equivalent to approximately 20 US Dollars.

*4 One patient who was a current smoker in each of the Group I and Group C were excluded due to missing data.

*5 The chi-square test was applied.

Supplementary Table S3 Odds ratios of tobacco-smoking status with binominal logistic regression analysis* between the Intervention site (Group I) and Control site (Group C) in Manila, by month of TB registration, from 2017 to 2018, the Philippines

	Odds Ratio (Intervention site vs. Control site)	95% Confidence Interval		z-value	*p*-value	E-value	
		from	to			Point estimate	Confidence Interval
Month 0 (n=2,153)	1.26	1.02	1.55	2.15	0.032	1.49	1.11
Month 2 (n=1,989)	0.43	0.30	0.62	-4.55	<0.001	4.06	2.61
Month 4 (n=1,936)	0.41	0.27	0.62	-4.26	<0.001	4.32	2.62
Month 6 (n=1,868)	0.45	0.30	0.68	-3.83	<0.001	3.89	2.32
Month 8 (n=1,643)	0.37	0.23	0.58	-4.25	<0.001	4.88	2.82
Month 12 (n=1,815)	0.44	0.29	0.66	-3.93	<0.001	3.95	2.38

TB: tuberculosis

Month 0: month at TB registration. Months 2 ~ 12: months from TB registration.

n: number of participants in the study each month from TB registration.

* Binomial logistic regression analysis adjusted to the site, sex, age category, patient category, TB type, patient delay, education level, marital status, occupation, regular incomes, monthly household incomes, and HIV & DM status. Current smoking status, i.e., the dependent variable, was assigned as 1 if the participant was either a current smoker or relapsed; otherwise, non-smokers, quitters, ex-smokers, or unknown was assigned as 0.

Supplementary Table S4 Odds ratios of tobacco-cessation status with binomial logistic regression analysis* between the Intervention site (Group I) and Control site (Group C) in Manila, by month of TB registration, from 2017 to 2018, the Philippines

	Odds Ratio (Intervention site vs. Control site)	95% Confidence Intervals		z-value	*p*-value	E-value	
		from	to			Point estimate	Confidence Interval
Month 2 (n=2,009)	1.92	1.51	2.45	5.30	<0.001	2.12	1.76
Month 4 (n=1,959)	1.83	1.44	2.33	4.98	<0.001	2.05	1.69
Month 6 (n=1,871)	1.97	1.54	2.52	5.44	<0.001	2.16	1.79
Month 8 (n=1,669)	2.05	1.58	2.67	5.34	<0.001	2.22	1.82
Month 12 (n=1,842)	1.90	1.49	2.44	5.09	<0.001	2.10	1.74

TB: tuberculosis

Month 0: month at TB registration. Months 2 ~ 12: months from TB registration.

n: number of participants in the study each month from TB registration.

* Binomial logistic regression analysis adjusted to the site, sex, age category, patient category, TB type, patient delay, education level, marital status, occupation, regular incomes, monthly household incomes, and HIV & DM status. Tobacco quitting status, i.e., the dependent variable, was assigned as one if the participant is a quitter; otherwise, current smokers, relapsed, non-smokers, ex-smokers, or unknown was assigned as 0.

Supplementary Table S5A Secondhand-smoking-at-home status (answered as yes) of the TB patients enrolled by month of TB registration, by the site in Manila, from 2017 to 2018, the Philippines

	Intervention site (Group I) (District I, Manila)	Control site (Group C) (District VI, Manila)	*p*-value *1
Month 0 (n=2,174)	461 (40.3%)	385 (37.4%)	0.163
Month 2 (n=2,096)	179 (16.3)	233 (23.4)	<0.001
Month 4 (n=1,979)	180 (17.8)	265 (27.4)	<0.001
Month 6 (n=1,967)	148 (14.5)	229 (24.2)	<0.001
Month 8 (n=1,919)	107 (10.8)	219 (23.6)	<0.001
Month 12 (n=1,878)	105 (10.8)	211 (23.3)	<0.001

TB: tuberculosis

Month 0: month at TB registration. Months 2 ~ 12: months from TB registration.

* Chi-square test was applied to test the statistically significant level of the proportions among the categories between the sites by month of TB registration.

Supplementary Table S5B Odds ratio of the secondhand-smoking-at-home status with logistic regression analysis* between the Intervention site (Group I) and Control site (Group C) in Manila, by month of TB registration, from 2017 to 2018, the

Philippines	Odds Ratio (Intervention site vs. Control site)	95% Confidence Intervals		z-value	*p*-value	E-value	
		from	to			Point estimate	Confidence Interval
Month 0 (n=2,153)	1.11	0.93	1.34	1.16	0.247	1.30	1.00
Month 2 (n=2,012)	0.71	0.57	0.90	-2.87	0.004	1.65	1.30
Month 4 (n=1,959)	0.63	0.51	0.80	-3.96	<0.001	1.82	1.49
Month 6 (n=1,871)	0.62	0.48	0.79	-3.92	<0.001	1.86	1.51
Month 8 (n=1,669)	0.65	0.49	0.85	-3.18	0.001	1.80	1.40
Month 12 (n=1,841)	0.44	0.34	0.57	-6.07	<0.001	2.39	1.97

TB: tuberculosis

Month 0: month at TB registration. Months 2 ~ 12: months from TB registration.

n: number of participants at each month from TB registration.

* Logistic regression analysis adjusted to the site, sex, age category, patient category, TB type, patient delay, education level, marital status, occupation, regular incomes, monthly household incomes, and HIV & DM status. Secondhand smoking status, i.e., the dependent variable, was assigned as one if assessed as secondhand-smoking at home, otherwise was assigned as 0.

Supplementary Table S6A TB treatment outcomes with binominal logistic regression analysis* by sites in Manila, TB patients registered from 2017 to 2018, the Philippines

	Intervention site (District I, Manila)	Control site (District VI, Manila)	*p*-value *
**TB Treatment Outcomes (n = 2,246)**			0.201
Successful (cured or treatment completed)	993 (85.0)	941 (87.3)	
Died	46 (3.9)	28 (2.6)	
Failed	8 (0.7)	6 (0.6)	
Lost to follow-up	101 (8.7)	78 (7.2)	
Not Evaluated / unknown	20 (1.7)	25 (2.3)	

TB: tuberculosis

Months 12: months 12 from TB registration.

n: number available for TB treatment Outcome.

* We applied the chi-square test to test the statistically significant level of the proportions among the categories between the sites.

Supplementary Table S6B Odds ratios of TB treatment success with logistic regression analysis[*1] by sites in Manila, TB patients registered from 2017 to 2018, the Philippines

	Odds Ratio (Intervention site vs. Control site)	95% Confidence Intervals		z-value	*p*-value	E-value	
		from	to			Point estimate	Confidence Interval
TB Treatment Success [*2] (n=2,195)	1.10	0.85	1.42	0.71	0.477	1.27	1.00

TB: tuberculosis

Months 12: months 12 from TB registration.

n: number available for TB treatment Outcome.

[*1] Binominal logistic regression analysis adjusted to the site, sex, age category, patient category, TB type, patient delay, education level, marital status, occupation, regular incomes, monthly household incomes, and HIV & DM status.

[*2] TB Treatment Success, i.e., the dependent variable, was assigned 1 if the outcome was either cured or treatment completed; otherwise, it was assigned 0.
